# How do medical institutions co-create artificial intelligence solutions with commercial startups?

**DOI:** 10.1007/s00330-025-11672-4

**Published:** 2025-06-03

**Authors:** Willem Grootjans, Uliana Krainska, Mohammad H. Rezazade Mehrizi

**Affiliations:** 1 Department of Radiology, Leiden University Medical Center, Leiden, The Netherlands; 2 Digital Business and Innovation Master, School of Business and Economics, Vrije Universiteit Amsterdam, Almere, The Netherlands; 3 KIN Center for Digital Innovation, School of Business and Economics, Vrije Universiteit Amsterdam, Amsterdam, The Netherlands

**Keywords:** Artificial intelligence, Interdisciplinary communication, Radiology, Technology transfer, Workflow

## Abstract

**Objectives:**

As many radiology departments embark on adopting artificial intelligence (AI) solutions in their clinical practice, they face the challenge that commercial applications often do not fit with their needs. As a result, they engage in a co-creation process with technology companies to collaboratively develop and implement AI solutions. Despite its importance, the process of co-creating AI solutions is under-researched, particularly regarding the range of challenges that may occur and how medical and technological parties can monitor, assess, and guide their co-creation process through an effective collaboration framework.

**Materials and Methods:**

Drawing on the multi-case study of three co-creation projects at an academic medical center in the Netherlands, we examine how co-creation processes happen through different scenarios, depending on the extent to which the two parties engage in “resourcing,” “adaptation,” and “reconfiguration.”

**Results:**

We offer a relational framework that helps involved parties monitor, assess, and guide their collaborations in co-creating AI solutions. The framework allows them to discover novel use-cases and reconsider their established assumptions and practices for developing AI solutions, also for redesigning their technological systems, clinical workflow, and their legal and organizational arrangements. Using the proposed framework, we identified distinct co-creation journeys with varying outcomes, which could be mapped onto the framework to diagnose, monitor, and guide collaborations toward desired results.

**Conclusion:**

The outcomes of co-creation can vary widely. The proposed framework enables medical institutions and technology companies to assess challenges and make adjustments. It can assist in steering their collaboration toward desired goals.

**Key Points:**

**Question** *How can medical institutions and AI startups effectively co-create AI solutions for radiology, ensuring alignment with clinical needs while steering collaboration effectively?*

**Findings** *This study provides a co-creation framework allowing assessment of project progress, stakeholder engagement, as well as guidelines for radiology departments to steer co-creation of AI.*

**Clinical relevance** *By actively involving radiology professionals in AI co-creation, this study demonstrates how co-creation helps bridge the gap between clinical needs and AI development, leading to clinically relevant, user-friendly solutions that enhance the radiology workflow.*

## Introduction

Radiology departments are increasingly interested in adopting artificial intelligence (AI) solutions [1], seeking to reduce workload and enhance quality of care [2, 3]. Despite promising research and pilot results, many AI solutions fail to deliver significant added value in clinical practice and are not widely implemented in daily operations [4].

Despite the large variety of available commercial AI applications, medical institutions struggle with the significant investment required to implement AI in clinical practice [5]. In particular, the misalignment between clinical needs and AI capabilities limits the added value of AI in an operational setting, reducing the incentive for medical institutions to invest in its implementation. This misalignment arises because AI applications are often developed by computer scientists and engineers who, despite their technical expertise, may have limited insight into the complexities of clinical practice and radiology workflows [6].

Furthermore, this misalignment is exacerbated by variability in workflows and information technology (IT) systems across hospitals, preventing seamless integrations of AI in clinical practice. Consequently, the implementation of commercial AI applications could be ineffective and may even hinder clinical practice.

To bridge the gap between AI development and real clinical needs, medical institutions and universities are encouraged to develop AI applications by themselves, also known as in-house development. In-house AI development offers control over the development cycle, as well as used training data, and envisioned workflow integration. While this approach leverages the required medical expertise, institutions often lack the experience to develop sophisticated clinical software applications, obtain legal approvals, and seamlessly integrate them into existing workflow systems.

Co-creation has been proposed as a viable approach through which medical institutions collaborate with industrial partners to combine expertise from both sides [6, 7]. However, co-creation dynamics vary widely, presenting risks for both parties, which should be carefully managed. The aim of this paper is to examine different types of co-creation processes and offer a practical framework for monitoring, assessing, and guiding the co-creation process as well as highlighting how partnerships can effectively develop AI applications tailored to clinical needs.

## Methods

### Available knowledge

Co-creation is effective for developing clinical solutions [8, 9]. By integrating the medical professionals’ input early in the development cycle, AI solutions are more likely to be clinically useful and accepted [10]. Furthermore, this approach ensures that AI solutions are sufficiently understood and transparent to the medical professionals [11]. However, co-creation is complex, involving cultural differences as well as both synergetic and diverging interests between medical and commercial parties. Success depends on alignment and careful management of expectations and projects [12].

Co-creation of AI applications often involves startups that are fast and agile, but often have limited resources and their priorities change based on market and investors’ demands [13]. While these differences can accelerate development by encouraging innovation and agility, they also increase the risk of sudden changes in startup priorities due to shifting market dynamics, such as the emergence of new disruptive technologies or a strong dependence on capital investors. On the other hand, medical institutions may struggle to provide the necessary data or expertise at critical development phases due to staffing changes, limitations in available resources, varying levels of clinician involvement, or evolving strategic goals within the medical departments.

Successful co-creation requires mutual ideation, experimentation, and validation throughout development, implementation, and post-implementation phases. Clinicians play an active role, from defining use-cases to validating newly created applications [7]. This process involves multiple rounds of validation and redesign, making co-creation an evolving journey shaped by continuous interactions between technology and medical parties. The complexity of co-creation relationships, particularly given the diversity of companies and types of AI applications, means there is no single template for collaboration. Decisions on stakeholder engagement, communication management, role definition, and collective decision-making must be made collaboratively, adjusted to each case, and revised along the way.

The novelty of AI solutions adds to the uncertainty, as medical institutions often lack experience with the clinical implementation of this technology. This can lead to friction and change of direction more frequently than with other more traditional technological innovations. Striking the balance between exploring new directions and adhering to the prior expectations and agreements is crucial in AI co-creation.

As a collaborative process between medical and technological parties, co-creation involves trade-offs between developing broadly useful products and tailoring solutions for specific use-cases. Medical institutions must also balance their current workflows and technological configurations with the need to adapt to new AI solutions. Depending on how these trade-offs are managed, co-creation can range from minimal engagement to tight collaboration, where both parties rethink assumptions and restructure workflows and technologies.

### Rationale: a relational framework of AI co-creation process

**Fig. 1:**
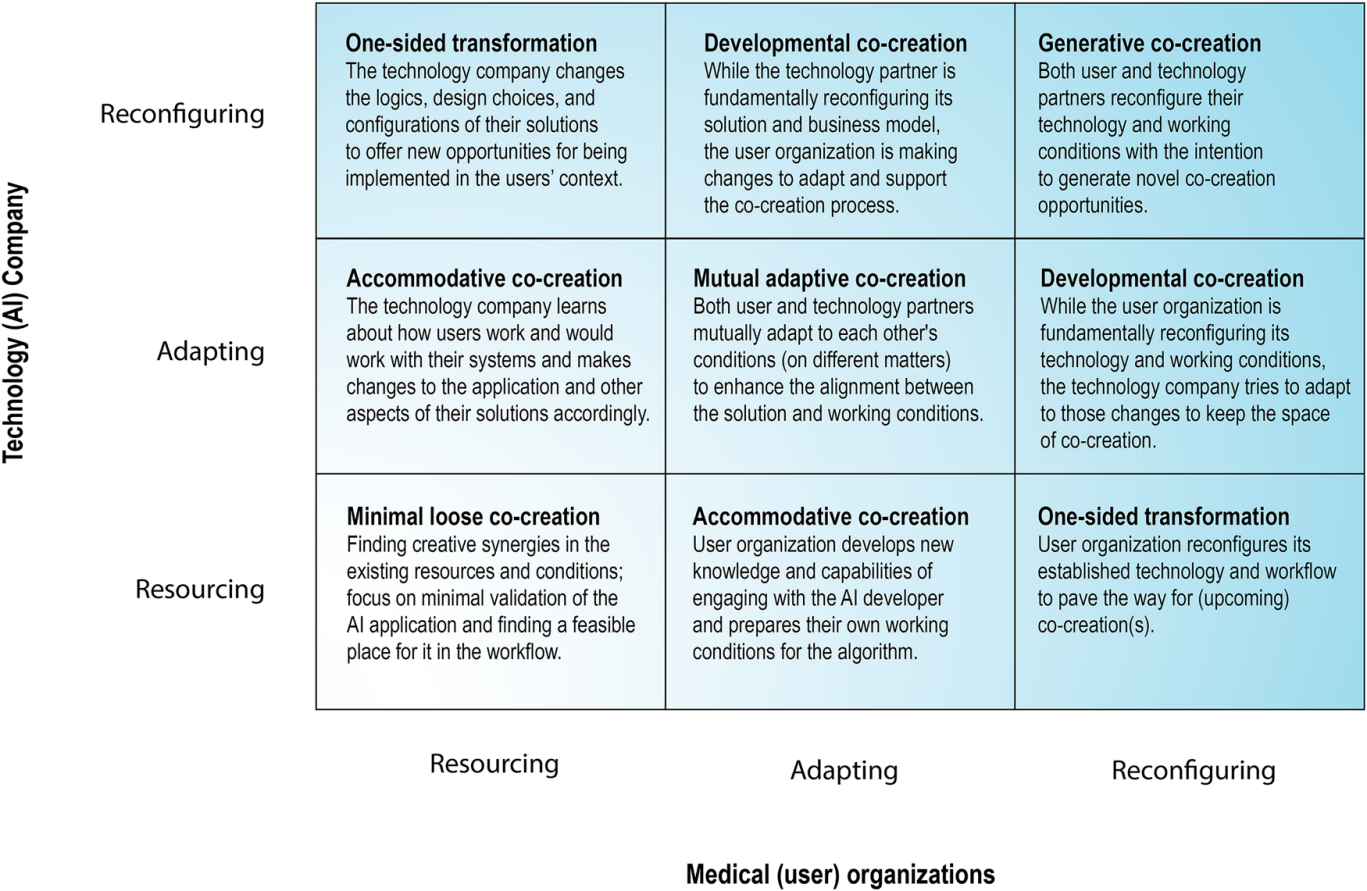
A relational co-creation framework. This dyadic map depicts the possible modes of engagement between the startup companies and medical institutions during the process of co-creation

To understand the co-creation process, we draw on strategic management literature and identify three modes of engagement [9, 14]:

1. Resourcing: allocating available technological, human, intellectual, financial, and physical resources to the co-creation project,
2. Adapting: modifying, complementing, or extending current strategies, decision, and resources
3. Reconfiguring: changing the existing assumptions, directions, and configurations of social and technological factors,

which can be seen over a continuum from limited to extensive scope and depth of changes required (see Table 1). In a dyadic co-creation relationship, these modes create a space of relational engagements (Fig. 1 shows that each co-creation process can be mapped as a trajectory in this space, across the nine relational engagement combinations).

**Table 1:** Three engagement modes in a co-creation process

Engagement mode	Examples
Resourcing involves allocating available technological, human, intellectual, financial, and physical resources to the co-creation project [23]. Key decisions include which resources to allocate, how to use them for co-creation tasks, and ensuring timely mobilization. Examples include assigning specific expertise, providing medical data or systems access, and offering financial resources. This mode poses the least risk to both the medical institution and the startup company.	- Adhering to the existing strategic directions - Maintaining fundamental design assumptions and choices related to systems - Decisions focused on selecting and allocating existing resources to co-creation tasks - Actions focused on mobilizing the existing resources for co-creation - Objectives: gaining efficiency in using the available resources
Adaptation refers to a moderate mode of engagement in the co-creation process through which an organization modifies, complements, or expands its current socio-technical conditions, such as increasing the capacity of and developing the technologies (e.g., processing capacity), or extending the range and scope of products and services that have been already developed, or strengthening certain domains of expertise that have been already in place. This mode of engagement keeps the strategic directions and fundamental assumptions, but further develops them within the established paradigm [14]. It involves more risk as it necessitates significant changes by the parties involved.	- Extending and strengthening the existing strategic directions - Extending and building further on the fundamental design assumptions and choices related to systems - Decisions focused on making changes that are aligned with the core business, fundamental directions, and established design and architecture of the systems - Objectives: expanding and deepening the current choices and actions for supporting co-creation
Reconfiguration involves changing the existing assumptions, directions, and configurations of social and technological factors, known as transformational change. It includes redesigning technological landscapes, revising organizational strategies, and altering role definitions. Unlike adaptation, reconfiguration entails fundamental changes to the status quo. This mode is high-risk but offers high rewards, requiring careful coordination and evaluation.	- Deviating from the existing strategic directions - Rethinking, questioning, and changing fundamental design assumptions and choices related to systems - Decisions focused on choices and actions that are fundamentally different from the past directions - Actions focused on going beyond the current practices and established setups or configurations of the systems and workflow - Objectives: breaking through the past orientations and making shifts and changes in the fundamental assumptions and design choices

### Context

We conducted a longitudinal, embedded case study [15] of Medica, a well-established academic hospital in the Netherlands, participating in three co-creation projects with AI startups: (1) BoneAI: a medium-sized European company focusing on developing deep learning (DL) models for analyzing musculoskeletal X-rays, (2) ScreenAI: a small European startup offering an AI solution for analyzing Chest X-rays, (3) ChestAI: a small European company offering chest-CT models for detecting, classifying, and quantifying lung pathologies. Medica, established in the second half of the 19th century with nearly 8000 staff, has been co-creating AI solutions with these startups since 2018. The co-creation process primarily involved the Radiology department forming an internal team of (1) medical specialists (radiologists and referring physicians) who provide insights into the medical context and evaluate the usability of the solution, (2) technical experts from radiology and central IT departments, (3) regulatory experts from security, privacy, and legal teams, and (4) workflow experts and department management. Additionally, Medica occasionally included their PACS provider in the team, particularly when integrating AI solutions into the radiology PACS viewer. This team worked with the technical teams of startups, their Chief Executive Officer (CEOs), Chief Technology Officer (CTO), product owners, and engineers.

By examining these cases, we analyzed how each co-creation process began and evolved. The consistent involvement of Medica across all cases allowed for comparing co-creation processes and outcomes.

### Data collection

We collected data through semi-structured interviews with stakeholders involved in co-creations. This included the core team at Medica and CTOs, CEOs, sales specialists, data engineers, and designers and document analysis from each startup. Additionally, participatory observation was employed, as one author, a member of Medica’s Radiology Department management team, was involved in the co-creation processes from the start. His role in managing relationships with AI companies and coordinating technical integrations provided rich insights into Medica’s internal steps and interactions with these companies. This data, collected through personal notes and meeting logs, offered an in-depth insider perspective [16]. To complement this insider view, independent researchers conducted semi-structured interviews with various informants from Medica and the startups. This was supplemented with archival data, including technical specifications and user manuals. This way, we captured the entire co-creation process, tracking the development of technology over the years, the decisions and actions taken by Medica and the startups, as well as the impacts on each party involved.

### Measures and analysis

We used confirmatory thematic analysis to code for engagement levels using a codebook from strategic management literature [17]. By juxtaposing it with the sequence of events and actions, we mapped each co-creation process onto our conceptual framework. The analysis included four steps:

1. Data compilation: compiling data on each co-creation process, mapping actions, decisions, and events using a “temporal mapping” strategy [18].
2. Temporal bracketing: identifying phases based on key decisions or changes in AI solution development and implementation following a “temporal bracketing” approach [18].
3. Framework mapping: mapping phases onto the co-creation framework (Fig. 1) to understand each co-creation journey.
4. Cross-case comparison: comparing cases to identify patterns in engagement trade-offs and their consequences (Table 2).

**Table 2:** Qualitative criteria to evaluate the co-creation process

Criteria	Detailed indicators
Co-creation process	- Timely interactions and harmonious exchanges and collaboration between the parties - Cost-effective and efficient use of resources for supporting the co-creation process - Progress and commitment: parties delivered on the promises and stayed committed to their tasks and decisions
Learning and knowledge gains	- New ideas and perspectives were developed and embraced by the parties - Novel practices and design choices and organizational mechanisms were developed - Ineffective and obsolete assumptions and choices and practices were questioned and rethought
Product innovation	- The focal AI solution was developed into a working solution for being implemented and used - New, effective use-cases are defined
Performance/productivity gains	- The AI solution is used in practice and creates productivity in the workflow, increases the quality of the decision and diagnosis, or any other positive outcomes for clinical workflow

### Ethical considerations

Informed consent was obtained from Medica and the participating startups to analyze data related to the co-creation process. External researchers were only given access to pseudonymized data, ensuring no sensitive information regarding patents or other non-disclosable content was shared. The startups reviewed the final manuscript before submission to ensure accuracy and protect their interests. No sensitive data pertaining to individuals or technologies was disclosed in a way that could harm the involved startups or Medica. This study did not involve analyzing any medical data, patient information, or data related to medical procedures. All ethical guidelines and standards were strictly followed to safeguard the privacy and confidentiality of all parties involved.

## Results

### Case 1: Quantitative vertebral morphometry

The first case involved the co-creation of a deep learning (DL) model for performing quantitative vertebral morphometry (QVM) on X-rays with an AI startup specializing in musculoskeletal cases (Table 3 and Appendix A1). Data collected included 856 emails exchanged with Medica (408 sent, 448 received, November 2018–February 2024), one legal agreement (master research agreement), biweekly meeting records (agendas, minutes, updates), project plans, data processing logs, training logs, site visit reports, shared documents (e.g., grant proposals, collaborative notes, summaries), interview transcripts (CTO, CEO, sales representatives and engineers, and evaluation reports, as well as two abstracts and three manuscript drafts). The innovation lead of Medica met with the CEO and CTO of BoneAI at an AI health event in 2018, where a few high-potential AI startups, including BoneAI showcased AI models for hip and knee analysis. At this event, the innovation lead’s role was to advise AI startups on aligning their clinical products with clinical practice. BoneAI was invited to attend an AI symposium at Medica where a Medica radiographer specializing in vertebral morphometry suggested to start a research project aimed at developing a related product. After initial contact, agreement from both Medica and BoneAI led to drafting of a research master agreement that established the framework for co-creation: Medica provided data and medical expertise, while BoneAI handled software development and legal certification, marking Medica’s first experience with MDR certification. Medica’s ethical and legal committee ensured proper data and service management. Medica shared 2500 cases for BoneAI to train a QVM model.

**Table 3:** Overview of co-creation processes and their outcomes

Case 1: Quantitative Vertebral Morphomet (moderate balanced engagement to unbalanced toward Medica)
Co-creation process
Motivation:
• The primary motivation for developing this AI application was to reduce the workload of radiographers by automating the annotation of lateral spine radiographs for QVM scoring in osteoporosis patients. Medica has recognized expertise in this domain.
Company characteristics:
• Founded in 2016, the company is based in Europe and employs approximately 28 full-time staff as of 2023.
• It has developed multiple AI applications for musculoskeletal X-ray analysis using deep learning.
Stakeholders:
• Medica: Musculoskeletal radiologist, specialized radiographer, endocrinologist, ethics committee and compliance officers, innovation lead (coordinating collaboration), medical data scientists, IT specialists, legal representatives (technology transfer office), and master students.
• BoneAI: Data scientists, software engineers, Chief Technology Officer (CTO), Chief Executive Officer (CEO), UX/UI designers, regulatory specialists, and product managers.
Evolution of co-creation over time:
• The co-creation process began with a balanced and adaptive approach, ensuring timely interactions and collaborative exchanges between Medica and BoneAI.
• Over time, Medica recognized the importance of a long-term expectation framework, ensuring commitment and progress despite evolving startup priorities.
• Despite the internalization of the QVM model, Medica remained committed to co-creation beyond this project, continuing other research collaborations.
Co-creation outcomes
Learning and knowledge gains:
• Medica recognized the need for a long-term expectation framework to better manage shifts in startup priorities, ensuring more stable and predictable collaborations. This was particularly important after the deprioritization of the co-creation project, where Medica learned that a sudden course change of the startup can stop a project.
• Medica gained expertise in structuring legal frameworks for intellectual property rights
• BoneAI refined its product strategy, learning how to align its AI applications with clinical workflows and integrate them into co-creation frameworks for more effective adoption in radiology departments.
• Despite the internalization of the QVM model by Medica, the broader co-creation framework remained active, stimulating ongoing research collaborations and new clinical AI initiatives.
Product innovation:
• The balanced and adaptive co-creation approach led to the development of an alpha prototype AI application, designed to support radiologists in automated MSK X-ray annotation.
• A generalized legal framework was established, streamlining the process for initiating new AI-driven projects in radiology while ensuring compliance with hospital and regulatory policies.
• A data exchange infrastructure was developed to enhance interoperability between AI applications and clinical systems, enabling efficient integration of future AI solutions.
Performance/productivity gains:
• Although the QVM model development was internalized by Medica, the collaboration continued generating research outputs, resulting in two scientific publications that contributed to AI advancements in musculoskeletal radiology.
• The partnership led to workflow efficiency improvements through the clinical implementation of two additional AI applications, significantly reducing annotation time for MSK X-rays in routine practice.
• By automating repetitive annotation tasks, the AI applications helped optimize radiologists' time, allowing them to focus on complex cases and improve diagnostic throughput, translating into better patient care and reduced workload strain on medical staff.

Case 2: Co-creating a chest X-ray solution (Unbalanced toward startup company, Medica following quick developments)
Co-creation process
Motivation:
• With a high volume of 23,000 chest X-rays per year, Medica recognized significant potential in automating aspects of chest X-ray reporting. Since 40–50% of chest X-rays are normal (i.e., showing no findings), such applications are expected to greatly enhance efficiency in the radiological workflow.
Company:
• Founded in 2017 and located in Europe, the company employs approximately 19 full-time staff (as of 2023)
• It developed multiple applications for X-ray (chest and MSK) as well as chest CT.
Stakeholders:
• Medica: Cardiothoracic radiologist, ethics committee and compliance officers, innovation lead (coordinating collaboration), medical data scientists and radiology IT specialists.
• ScreenAI: Data scientist, software engineer, chief technology officer (CTO), chief executive officer (CEO), product manager
• PACS: Software engineers and applications specialists
Evolution of co-creation over time:
• The collaboration followed a highly adaptive and agile approach, with Medica actively driving reconfigurations in the AI company, which responded swiftly.
• The engagement evolved over time, with the AI company progressively taking a more proactive role, while Medica adjusted to these developments
• The partnership remained strong, leading to continuous adaptation and refinement of the co-creation process.
• ScreenAI formally participated in new research projects as a consortium partner
Co-creation outcomes
Learning and knowledge gains:
• Medica gained expertise in seamlessly integrating AI solutions into its clinical workflow, enabling continuous refinement of use-cases and value propositions to better align with real-world radiology needs.
• ScreenAI reassessed and pivoted from its initial use case, shifting its focus to automated screening of normal X-ray cases, allowing radiologists to dedicate more time to complex cases.
• ScreenAI developed a new dashboarding application, transforming its tool into a real-time quality feedback system, helping radiologists improve report consistency and accuracy.
• The co-creation process provided both partners with a deeper understanding of how AI tools can be optimized for radiology workflows, ensuring better alignment with hospital operations and staff needs.
Product innovation:
• The AI solution was successfully implemented in clinical practice, allowing for automated filtering of approximately 15–20% of normal chest X-ray cases while maintaining 99.8% sensitivity, reducing unnecessary manual reviews.
• The AI application was progressively integrated into PACS, achieving seamless workflow integration, ensuring minimal disruption to radiologists' daily practice.
• An automated natural language processing (NLP) system was developed and deployed for report analysis, improving the efficiency of structured reporting in radiology.
• A quality check system was implemented to standardize radiological reporting, reducing diagnostic variability and enhancing decision-making accuracy.
Performance/productivity gains:
• The AI-based normal/abnormal classification of chest X-rays led to efficiency gains, significantly reducing time spent on reviewing normal cases, allowing radiologists to prioritize complex imaging studies.
• The collaboration extended beyond the original project scope, resulting in the exploration of additional AI applications in radiology, demonstrating the scalability and adaptability of AI solutions.
• Several scientific publications and social media reports were shared, disseminating insights from the co-creation process, contributing to awareness and adoption of AI in clinical practice.
• Scientific manuscripts were drafted, furthering knowledge-sharing and advancing AI integration in radiology.

Case 3: Co-creating a chest-CT analysis solution (Balanced from lightly to moderate and eventually heavily engaged from both sides)
Co-creation process
Motivation:
• The need to follow up on numerous chest-CT scans for lung patterns, including pulmonary nodule assessment, highlighted the potential for automation. The AI solution was designed as a comprehensive suite for chest-CT analysis, aimed at delivering quantitative reports and significantly improving workflow efficiency.
Company:
• Founded in 2016 and located in Europe, the company employs approximately four full-time staff (as of 2023) and is primarily driven to develop dedicated AI solutions for analyzing chest-CT images and diagnosing various lung pathologies.
Stakeholders:
• Medica: Cardiothoracic radiologists, ethics committee and compliance officers, innovation lead (coordinating collaboration), medical data scientists and radiology IT specialists.
• ChestAI: Data scientist, software engineers, CTO, CEO, product manager, partnerships/relation manager, research coordinator.
• PACS: Software engineers and applications specialists.
Evolution of co-creation over time
• The co-creation process was balanced and highly engaged, with both Medica and ChestAI actively participating in reconfigurations and investing in long-term collaboration built on mutual trust and respect.
• Both parties demonstrated commitment and progress by consistently delivering on their tasks and adapting to new insights gained throughout the process.
• The collaboration resulted in efficient resource utilization, as both teams worked closely with PACS providers and radiologists to seamlessly integrate AI into existing workflows.
Co-creation outcomes
Learning and knowledge gains
• Medica gained extensive experience in implementing cloud-based AI systems, allowing for scalable, remote access to AI-driven radiology solutions, optimizing workflow flexibility.
• Medica also developed expertise in engaging radiologists in structured feedback processes, ensuring that AI solutions were refined based on real-world clinical needs.
• Medica and ChestAI learned that continuous long-term optimization of the application, with careful attention to small details, is essential for ensuring that the AI application meets clinical requirements.
• ChestAI redefined its application and value proposition, improving its ability to align AI tools with radiological workflows, leading to higher clinical adoption and usability.
• The co-creation process established new organizational mechanisms for AI deployment, enabling seamless integration of automated feedback loops into radiology workflows.
• Both organizations adopted novel strategies for AI deployment, ensuring that future AI solutions could be sustainably integrated and continuously refined.
Product innovation
• The co-creation process resulted in the development of a cloud-based AI solution, enabling real-time access to image analysis tools across multiple radiology workstations, enhancing workflow efficiency.
• The AI application was seamlessly integrated into the clinical workflow, achieving deep PACS integration in later iterations, ensuring minimal disruption to radiologists' existing processes.
• Multiple pilot implementations were launched, allowing for continuous refinement and optimization of the AI tool based on real-world use-cases, leading to workflow improvements for both Medica and ChestAI.
• The AI solution was tested across different imaging modalities, demonstrating its adaptability and potential for broader clinical application, making it a scalable solution for AI integration in radiology.
Performance/productivity gains
• The collaboration led to the launch of several research projects, extending the scope of AI-driven imaging applications, ensuring continuous innovation in radiology AI.
• The initiative facilitated the submission of multiple grant applications, securing additional funding for future AI developments and supporting further technological advancements.
• The AI system reduced radiologists’ manual workload by automating routine imaging tasks, allowing faster turnaround times and improving diagnostic efficiency.
• Findings from the co-creation efforts were disseminated in scientific publications and social media channels, enhancing the visibility of AI-driven radiology advancements and supporting broader AI adoption in medical imaging.

BoneAI developed an alpha prototype which was tested on 150 cases, revealing potential despite accuracy issues, necessitating new training data and sanity checks to be integrated into the software. However, BoneAI shifted focus to other products due to resource limitations, and the COVID-19 pandemic complicated Medica’s involvement at that time.

**Fig. 2:**
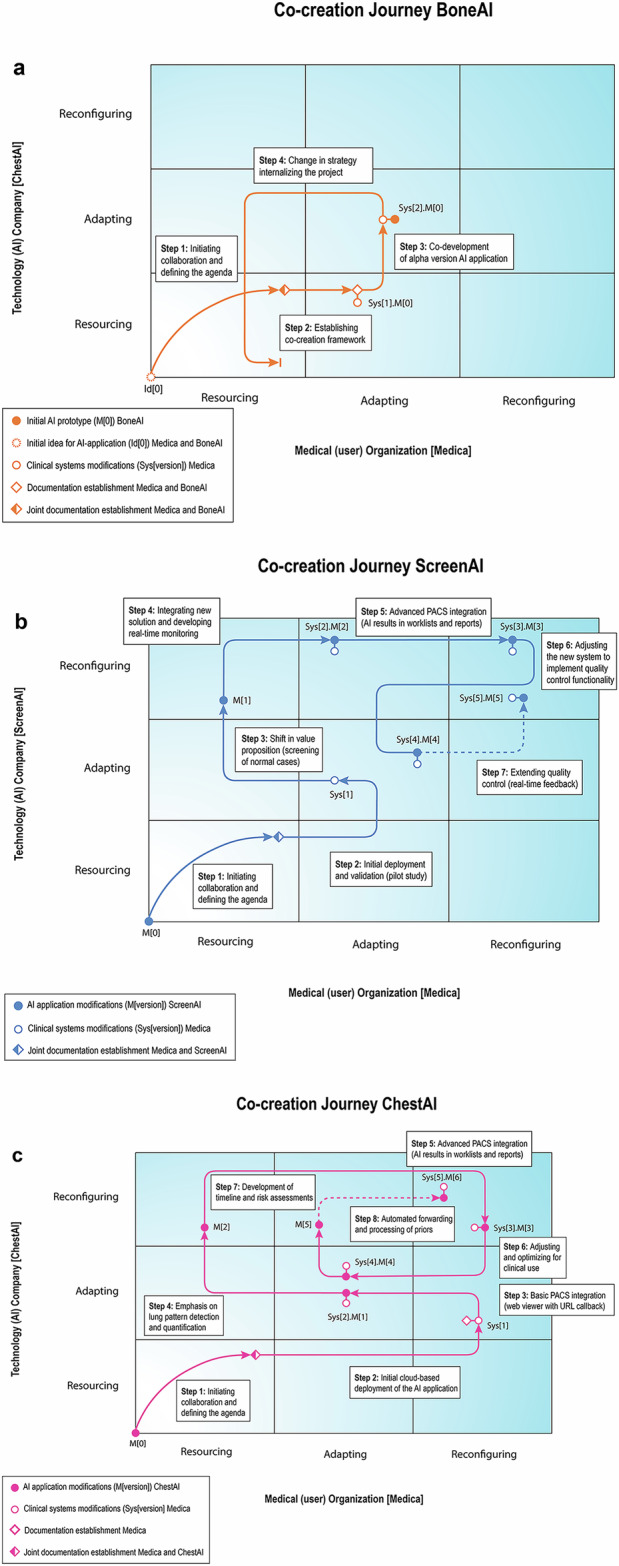
Co-creation processes between Medica and BoneAI (**a**), ScreenAI (**b**), and ChestAI (**c**). Each co-creation journey is mapped onto the proposed relational framework, illustrating the progression of collaboration. Key artifacts, including prototypes and documentation, as well as their modifications, are indicated at each step

Despite these challenges, Medica began implementing other BoneAI products and sought research grants and in-house resources to stimulate the development of the QVM model. Eventually, BoneAI officially deprioritized the co-creation project. The collaboration framework remained intact through other research projects. Consequently, the co-creation process was postponed and, despite promising results, the AI solution was only limitedly developed. However, it provided important learning points for Medica regarding establishing legal frameworks for intellectual property rights and for BoneAI in recognizing the importance of a long-term product strategy for co-creation collaborations (Fig. 2a).

### Case 2: Co-creating a chest X-ray solution

The second case involved modifying a commercially available AI model for detecting several pathologies on chest X-rays (Table 3 and Appendix A2). Initially, this model was intended for triaging and automated reporting. Data collected for this case included 1111 emails exchanged with Medica (360 sent, 751 received, November 2018–February 2024), one legal agreement, biweekly meeting records (agendas, minutes, updates), project plans, data processing logs, training logs, shared documents (e.g., grant proposals, collaborative notes, summaries), interview transcripts (CTO, CEO, engineers), and evaluation reports. In October 2018, ScreenAI connected with Medica through a Spanish Medtech network to test their product and explore its clinical value. The department of Radiology assigned this project to the innovation lead at Medica. Eager to integrate AI applications, Medica collaborated with ScreenAI to draft a collaboration contract.

After the initial deployment, a research pilot focused on validating 100 cases, which showed accuracy issues such as missed fractures. Following further exploration and feedback from Medica and other centers, ScreenAI changed the value proposition of their product to provide a “normal/abnormal” classification of chest X-rays. The revised solution was implemented, tested, and validated at Medica.

To maximize clinical value in terms of reducing the workload, focus shifted to seamlessly integrating the AI-generated results into the PACS viewer. This collaboration between the PACS vendor, ScreenAI, and Medica enabled the product to be fully integrated into PACS worklists and reporting systems. With ScreenAI developing automated monitoring methods, Medica utilized their in-house data platform to retrieve radiological reports from the electronic medical records (EMR) to be analyzed by ScreenAI’s natural language processing (NLP) model to create a real-time monitoring dashboard.

Medica and ScreenAI worked together to resolve initial bugs, integrate new features, and refine their data platforms. A quality monitoring system flagged discrepancies between AI and human observations, creating a feedback loop between radiologists and the AI. Future phases involve redefining the AI solution for real-time feedback and automated reporting, transforming it into an active agent. This raised new ethical and legal considerations and AI’s role in clinical workflows, currently being investigated at Medica (Fig. 2b).

### Case 3: Co-creating a chest-CT analysis solution

The third journey involves the co-creation of an AI solution initially designed as an image-based search tool for radiologists (Table 3 and Appendix A3). Through collaboration with institutions like Medica, it evolved into a platform for detection, quantification, and follow-up analysis of chest-CT images. For this case, the collected data included 1601 emails exchanged with Medica (497 sent, 1104 received; December 2018–February 2024), a legal agreement, documentation on data processing, privacy, and security, records from monthly meetings (agendas, minutes, and updates), project plans, training logs, shared documents (e.g., grant proposals, clinician feedback, collaborative notes, and summaries), interview transcripts (CTO, CEO, engineers). In 2018, ChestAI and Medica began a research collaboration after meeting at a medtech event, focusing on evaluating the search function with feedback from Medica. The innovation lead at Medica convened key stakeholders to review legal, privacy, and data processing documentation for the initial implementation.

Implementing this AI application required Medica to redesign its IT systems for cloud-based applications, overcoming initial skepticism from the IT department and addressing necessary security checks. Medica integrated the solution as a web application connected to its PACS viewer using a URL-based callback functionality, with automated forwarding rules for image retrieval and processing.

Initial findings showed limited value for Medica’s purposes (e.g., reducing the workload or examination time), prompting a shift to focus on the detection and quantification of lung patterns. This became particularly relevant during the COVID-19 pandemic for chest-CT reporting. Medica provided continuous feedback, leading to deeper and a less disruptive integration with PACS for seamless operation and better data transfer. Based on experience from integrating ScreenAI (Case 2), a similar PACS integration was pursued in collaboration with Medica, ChestAI, and the PACS vendor.

Further discussions led to the tuning of the AI solution and seamless integration of AI results in radiological reports. Feedback from Medica and other institutions emphasized the need for chronological analysis of multiple cases and risk classification of pulmonary nodules. In response, Medica focused on data organization, automated forwarding, and involving radiographers in preparing and analyzing cases with AI, ensuring smooth implementation of timeline evaluations (Fig. 2c).

## Discussion

As we see across the cases, co-creation of AI solutions requires continuous interaction between the startup and medical institution. The variety of scenarios described underscores the unpredictability of the co-creation process, which both parties must embrace. Success relies not only on the final product but also on the process, stakeholder engagement adapting legacy systems, workflows, and use-cases [19]. Simply allocating resources without a clear strategy, adaptation and reconfiguration hinders progress. Co-creation involves not just adding new knowledge but also rethinking established ideas. This dual process of learning and unlearning is crucial since many design choices and implementation strategies must be collaboratively identified, as many ideas and use-cases cannot always be foreseen in advance [20, 21].

Co-creation in itself is not new, and many medical institutions are, or have been, engaged in various degrees with industrial partners to develop new technology. However, typical co-creation relationships are with larger established companies that bring a certain degree of stability to the co-creation projects. Co-creation with startups is more dynamic and changes over time with different efforts, resources, and modes of engagement. The process is influenced by cultural differences, mutual interests, and the capacities of both startup companies and medical institutions [22].

### Management of co-creation and partner selection

Managers can assess their co-creation process based on different engagement scenarios and determine appropriate measures. These measures depend on the project’s stage, resource availability, and evolving dynamics between partners. To maintain momentum and alignment, a structured project team is essential, with an innovation lead from the medical institution coordinating the collaboration and a collaboration lead from the startup (usually the product manager or CTO) facilitating engagement. Together, they guide a team of clinicians, IT specialists, and engineers, ensuring that co-creation efforts remain focused and efficient.

Different engagement strategies support various phases of co-creation: in early stages, frequent touch points such as site visits, symposia, and radiological conference sessions help establish collaboration. As projects mature, periodic strategic meetings reinforce alignment and adaptation. These interactions also create opportunities to identify potential new partners, expanding the institution’s network for future co-creation initiatives.

Strategic partner selection is crucial for sustainable collaboration. Choosing partners based on radiological subspecialty or imaging modality ensures alignment with institutional expertise and needs. This enables long-term relationships, allowing for deeper co-development of multiple applications while maintaining an efficient collaboration structure. Although partner selection is not explicitly embedded in the proposed framework, identifying the right co-creation partner depends on cultural fit, future plans, and working methods. At Medica, partner selection followed a case-by-case strategic alignment approach, considering the specific needs of different radiological subspecialties rather than a fixed framework. While there was no strict project prioritization, openness to collaboration and alignment with institutional objectives played a key role in selecting companies. Pilot projects served as a valuable initial test to assess compatibility before expanding collaborations.

### Co-creation scenarios

The co-creation cases revealed several key lessons for optimizing AI integration in clinical practice. Early and continuous clinician involvement ensured alignment with clinical workflows and encouraged progressive acceptance of AI solutions. Iterative prototyping helped shift mindsets, enabling stakeholders to engage with evolving technology and adjust gradually to new developments. Additionally, having an innovation lead from the medical institution and a collaboration lead from the startup was essential for maintaining structured coordination and project momentum. The importance of long-term optimization with careful attention to small details became evident, as these refinements were critical for ensuring AI applications met clinical requirements.

For future co-creation initiatives, several improvements could enhance efficiency and impact. Clearer regulatory roadmaps and early compliance planning would help mitigate delays in clinical implementation. Structured funding and resource allocation could ensure sustainability beyond the pilot phase. Early discussions on intellectual property and commercialization would align expectations and minimize potential friction. Finally, stronger integration of co-creation steps into institutional workflows could streamline processes and improve long-term adoption.

It is important to realize that in the proposed relational framework, each mode of engagement has its purpose, and there is no requirement to pursue specific modes. For example, while reconfiguration can bring large gains, excessive reconfiguration can be risky and disruptive. Conversely, getting stuck in the resourcing phase limits the exploration of novel technology and outcomes. Therefore, a balanced approach combining resourcing, adaptation, and reconfiguration is essential for a sustainable co-creation relationship.

**Fig. 3:**
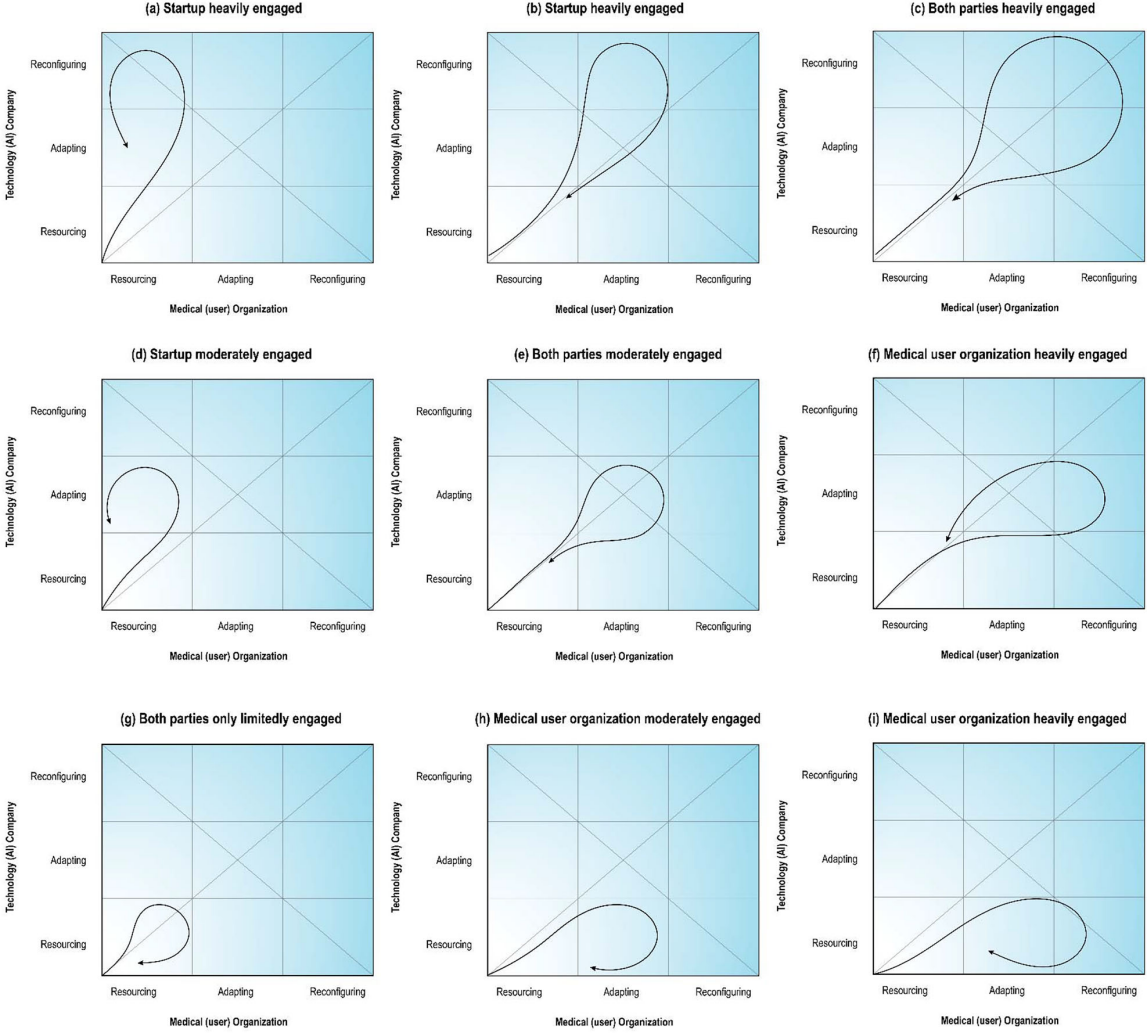
Balanced and unbalanced co-creation scenarios (**a**–**i**). Balanced scenarios (**c**, **e**, **g**) follow the diagonal line, where both parties engage similarly or return to this state over time. Unbalanced scenarios show a skewed distribution, with engagement favoring either the medical organization (**f**, **h**, **i**) or the technology company (**a**, **b**, **d**). In highly unbalanced cases, if only the medical organization drives reconfiguration (**i**), it may lead to breakthrough clinical changes without technology supporting these changes, whereas if only the technology company drives changes (**a**), AI solutions often require redesign to fit clinical needs. When the medical institution leads reconfiguration while the company mainly adapts (**f**), fundamental shifts in practice and technology may follow. Conversely, if the company drives major changes while the medical institution adapts (**b**), engagement becomes asymmetrical where solutions might not be completely embraced by clinicians. The highest level of mutual engagement and innovation occurs when both parties make major changes and reconfigure and design their systems (**c**), often prompted by breakthrough technologies or novel use-cases

Furthermore, this framework complements other project management strategies to yield desirable outcomes. Although this work followed three co-creation cases, a range of possible scenarios exists depending on the relative engagements by medical institutes and startup companies (Fig. 3).

### A strategic tool

The proposed framework provides a strategic tool for mapping, monitoring, and evaluating co-creation scenarios by tracking mutual engagement. It helps in choosing appropriate proactive measures through which co-creation can be strategically managed, such as setting new milestones or adjusting collaboration methods to ensure alignment and project progress. Reactive measures might involve adjusting resource allocations, realigning project goals, or revisiting partnership agreements to address unforeseen challenges. In case of unexpected events that halt co-creation, the framework provides a tool to reassess the mutual collaboration and bring the co-creation back on track. Medical institutions and startup companies can use this framework to assess and strategize their engagements across multiple projects. For example, a medical institution might reconfigure its workflow to integrate applications co-developed with various startups, while startups adjust their engagement based on available resources and client needs.

### Limitations and future research opportunities

Our findings are limited to the specific cases examined due to the qualitative nature of this study. Further research should explore other forms of co-creation, particularly collaborations with large, established companies that have greater resources but may be less agile. Future studies could examine how different engagement levels influence processes, learning, innovation, and performance. Additionally, understanding how medical institutions manage their co-creation portfolios is crucial, as engagement in one project may impact others. For instance, institutions may prioritize strategic collaborations when resources are limited, while technology companies must refine learning strategies based on past co-creation experiences. This study focused on publicly funded medical services, where co-creations are not directly tied to financial incentives for healthcare services delivered. In healthcare systems where financial factors play a stronger role, different co-creation scenarios may emerge, offering opportunities for further empirical study.

## Conclusions

As radiology departments integrate AI solutions into their clinical workflows, they inevitably engage in co-creation processes with the developers of these technologies. It is crucial that this co-creation occurs through balanced and robust engagement among the parties, continuously evaluated and adjusted in response to new ideas and emerging challenges. Our study is a foundational step in providing a strategic framework for assessing, monitoring, and guiding this uncertain process. Therefore, we propose that the focus should not be solely on the development of AI products but also on the co-creation process itself, the learning accrued by the parties involved, and the enhancements made to clinical workflows and technologies.

## Supplementary information

ELECTRONIC SUPPLEMENTARY MATERIAL
