# Supplementary material for: How do medical institutions co-create artificial intelligence solutions with commercial startups?
Source: Eur Radiol. 2025 Jun 3;35(12):7796–808. doi: 10.1007/s00330-025-11672-4 (PMC12634776; doi:10.1007/s00330-025-11672-4)
Supplement: Supplementary file 1 — ELECTRONIC SUPPLEMENTARY MATERIAL [file 330_2025_11672_MOESM1_ESM.pdf]

How do medical institutions co-create artificial intelligence solutions with commercial startups?
ELECTRONIC SUPPLEMENTARY MATERIAL

Detailed co-creation processes

Table A1 : Co-creation of quantitative vertebral morphometry with BoneAI

Co-creation step (events)	Medica moves	AI startup moves
**Step 1 (August - September 2018): Initiating the co-creation project as a joint experimentation on the preliminary products**<br><br>● **September 2018**: Initial meeting between company owners and Medica during the Medtech's health event to support promising AI startups.<br>● **November 2018**: The company attended the event hosted by Medica to showcase its products in an internal research event and promote them to clinicians, receiving feedback on two already available AI applications.<br>● Clinicians, including a radiographer from nuclear medicine and experts in bone density and osteoporosis, provided feedback and asked BoneAI if they could develop a solution to assist with the clinical practice of osteoporosis patients.<br>● BoneAI accepted the opportunity, and a contract was established to co-create the project between BoneAI and Medica.<br><br>Stakeholders and time commitment: Radiologists: ~0.5 hours/week (providing clinical feedback on AI feasibility), radiographers: ~1 hour/week (evaluating imaging workflow integration), innovation lead: ~2 hours/week (coordinating early-stage collaboration and strategic alignment), legal & compliance officers: ~1 hour/week (initial contract discussions and research agreement setup), collaboration lead BoneAI.	*Resourcing*<br><br>● Utilized its network and connections with companies and internal expertise (medical and AI experts) to explore the possibilities of further developing the AI solution.<br>● Leveraged its existing pool of experts and resources, maintaining the current context of AI research.<br>● Shared data resources and set up an automated infrastructure to retrieve, export, and curate data for further training the algorithm	*Resourcing*<br><br>● Committed to using its current models and expertise for training the model.<br>● Shared its expertise (intellectual capital) with Medica for potential future commercialization.<br>● Investigated the market potential of the solution.<br>● Committed internal expertise to the initial exploration.
**Step 2: (March 2019): Establishing the framework and preparing for the co-creation**<br><br>● Defining and approving a master research agreement to serve as an enabling framework for various projects during the collaboration.<br>● Agreeing that Medica provides content, data, and medical expertise while BoneAI handles software and legal certification of the product.<br>● With the new MDR (Medical Device Regulation) almost in place, BoneAI anticipated the	*Adaptation (major)*<br><br>● Extending knowledge and practices on engaging in data sharing legally and ethically with external developers.<br>● Expanding existing research contracts and practices to create a	*Resourcing*<br><br>● Mobilizing current technological infrastructure and resources, as well as expertise, for defining the collaboration framework.

certification process to meet the MDR requirements.<br>● The ethical and legal committee of Medica developed a collaboration framework addressing data sharing, anonymization, and value return for providing resources.<br><br>Stakeholders and time commitment: Radiologists: ~0.5 hours/week (input on clinical feasibility and workflow integration), radiographers: ~1 hour/week (assessing practical implementation and usability), innovation lead: ~2 hours/week (coordinating legal, technical, and strategic aspects of co-creation), legal & compliance officers: ~2 hours/week (drafting and approving research agreements, ensuring regulatory compliance), collaboration lead BoneAI.	collaboration framework for multiple research projects.<br>● Envisioning returns on joint development and data sharing with BoneAI, which was a new concept for Medica.	
**Step 3: (March 2019 end of the year) Co-developing alpha version of the application**<br><br>● 2,500 cases were collected by Medica and shared with BoneAI for training the model. Medica set up a data retrieval, export, and curation infrastructure, using DICOM metadata to sort, filter, and group files. This required technical modifications to their search and archiving system to extract the cases, as well as manual testing and fine-tuning scripts.<br>● A full master student was assigned to the project by Medica.<br>● BoneAI developed a prototype using Medica's data, capable of setting landmarks for performing quantitative vertebral morphometry on spinal X-rays.<br>● Medica tested the results based on 150 cases, qualitatively checking the cases where AI performed well, medium, and poorly.<br>● Medica examined the effect of using the solution on time reduction and user experience of the radiographers as a form of validation study.<br><br>Stakeholders and time commitment: Radiologists: ~0,5 hours/week (prototype testing of AI), radiographers: ~1.5 hours/week (user experience and workflow assessment), innovation lead: ~2 hours/week (coordination and project oversight), IT Specialists: ~5 hours/week (data retrieval, infrastructure modifications), master's student: Dedicated full-time involvement in supporting data collection and analysis, AI engineer BoneAI, collaboration lead BoneAI.	*Adaptation*<br><br>● Reverse-engineering clinical viewing system in a test environment to integrate AI results into the viewer used in clinical routine, thereby learning technical expertise on workflow systems.<br>● Expanding knowledge on how to validate new forms of AI solutions through effective validation experiments.	*Adaptation*<br><br>● Adapting their systems to create outputs of the algorithm in XML formats (previously used PDF) to make it readable by Medica's viewing systems.
**Step 4 (September 2019- January 2023): Intending to improve the model but facing delays and changes in the strategy**<br><br>● The next step was to retain the algorithm within	*Resourcing (actively)*<br><br>● Mobilized their relations	*Resourcing* (*limitedly*)<br><br>● Reconsidered

the 10cm around the Spine S to avoid detecting outside the region of interest (September 2019).<br>• BoneAI intended to work on this development but faced limited resources, admitting that it lacked sufficient resources for this project. This was mainly due to reallocating resources to another AI product requiring MDR accreditation (March 2020).<br>• Due to the COVID-19 pandemic, Medica was also limited in its involvement in the co-creation but continued using other AI products of BoneAI, keeping the general research and collaboration framework open (March 2020 - March 2021).<br>• BoneAI officially indicated that the co-creation project was no longer a strategic priority (March 2021).<br>• Medica attempted to obtain research grants together with BoneAI's grant consultant for developing the algorithm internally (unsuccessful in obtaining the grant) (March 2021 - March 2022).<br>• Medica explored other avenues (in-house development) or collaborations to develop the desired product (March 2022 - end of 2022).<br>• Medica began in-house development, setting up internal infrastructure required for training DL models (January 2023).<br><br>Stakeholders and time commitment: Innovation lead: ~2 hours/week (efforts to sustain collaboration and explore funding opportunities), legal & compliance officers: ~1 hour/week (negotiating and restructuring agreements as BoneAI shifted priorities), research grant consultant: Intermittent involvement (supporting grant application process, March 2021–March 2022), Medica IT specialists: ~3 hours/week (evaluating internal infrastructure for in-house AI development), PhD student for developing in-house AI model.	and legal framework with BoneAI to keep the co-creation project alive.<br>• Actively obtained new resources for advancing technological developments internally.	the allocation of technological and human resources to the project.<br>• Passively leveraged their relational resources with Medica to find a compromise, offering Medica to take the lead and own the new developments of the product.

Table A2 : Co-creation of Chest X-ray application with ScreenAI

Co-creation step (events)	Medica moves	AI startup moves
**Step 1 (October. 2018 - end of the year) Initiating the collaboration and setting the agenda**<br><br>● **October 2018**: ScreenAI connected with Medica through a Spanish networking organization.<br>● ScreenAI expressed its interest in testing their product at Medica and conducting research to evaluate its practical value.<br>● Medica expressed interest in benefiting from AI in clinical practice and participated in determining the product's additional value.<br><br>Stakeholders and time commitment: Radiologists and residents: ~0.5 hours/week (providing clinical insights and evaluating AI feasibility), innovation lead: ~2 hours/week (coordinating initial collaboration and defining project scope), legal & compliance officers: ~1 hour/week (reviewing legal and ethical considerations for co-creation), AI startup technical team (time allocated for defining test parameters and system evaluation).	*Resourcing*<br><br>● Leveraged data, expertise resources, and patient pool for co-creation.<br>● Committed a team of experts to define the collaboration framework.	*Resourcing*<br><br>● Used their application at other institutions for evaluation at Medica.<br>● Employed technical knowledge and personnel to define the required tests.
**Step 2: (February 2019 - end of the year): First implementation, tuning, and validation of the AI application**<br><br>● Installation and Testing: Installation and testing of the AI application at Medica involved contracting and security discussions. Issues were resolved, and software adaptations were made.<br>● Initial Research Initiatives: Conducted initial research with ScreenAI, focusing on the algorithm's performance using Medica's data.<br>● Technical Adjustments: Made technical adjustments to facilitate image forwarding and result integration into PACS.<br>● Validation: Read and validated 100 cases analyzed by the algorithm, identifying issues (e.g., missed fractures) and improving AI model performance.<br>● Critical Assessment: ScreenAI reassessed the value proposition, shifting focus to making reports with a complete differential diagnosis.<br><br>Stakeholders and time commitment: Radiologists and residents: ~1 hour/week (validating AI results, identifying issues in algorithm performance), IT Specialists: ~3 hours/week (technical adjustments for image forwarding and PACS integration), innovation Lead: ~2 hours/week (overseeing implementation and security discussions) ScreenAI AI Engineers (dedicated time for refining model performance based on clinical validation feedback).	*Adaptation*<br><br>● Extended internal systems and practices to forward images to ScreenAI.<br>● Enhanced security and contracting practices.<br>● Developed validation knowledge and recognized AI's limited benefits in the current context.	*Adaptation*<br><br>● Developed technical methods to integrate results into PACS (GSPS overlays).<br>● Expanded knowledge of AI limitations based on Medica's findings.<br>● Advanced understanding of clinical usability and solution value.

**Step 3: (2020) Shift in the value proposition and logic of the solution**<br><br>● Feedback and Discussions: Feedback from radiologists and other clients indicated the model's benefit in screening out "normal" cases.<br>● Redesign: ScreenAI redesigned the value proposition and presentation of AI results to indicate "normal/abnormal" instead of providing a differential diagnosis.<br>● Active Involvement: Medica offered insights and thought along on developing the algorithm in line with the new value proposition, mainly by allocating technical resources and clinical insights.<br><br>Stakeholders and time commitment: Radiologists and residents: ~0.5 hours/week (feedback on AI's clinical value and usability), innovation lead: ~2 hours/week (aligning project direction with updated value proposition), IT specialists: ~1 hour/week (assessing technical feasibility of workflow changes), ScreenAI AI engineers (dedicated effort in redesigning the AI model).	*Resourcing*<br><br>● Offered insights and thought along on developing the algorithm in line with the new value proposition.<br>● Allocated research time and clinical insights.<br>● Did not make changes to current practices or systems.	*Reconfiguration*<br><br>● Shifted the AI solution's fundamental concept and use-case from "differential diagnosis" to "normal case screening".<br>● Rethought the value proposition, user perception of outcomes, and workflow integration.
**Step 4 (mid 2020- mid 2021): Integrating new solution and developing automated system for performance monitoring**<br><br>● New Configuration and Testing: Implemented the new AI solution configuration at Medica and tested it to increase sensitivity, ensuring no false negatives.<br>● NLP Model Development: ScreenAI developed a natural language processing (NLP) model to analyze radiological reports for quality checks and to establish ground truth for validation. Medica provided translations to process Dutch reports, enabling performance checks and tuning of the AI model.<br>● Monitoring System: Tuned the AI model to multiple datasets across different hospitals, guiding implementation.<br>● Dashboard Creation: Created a dashboard for live monitoring of several aspects of the ChestAI application over time.<br>● Bug Fixing and Feature Addition: Medica adopted the system, helped fix bugs, and added new features. They modified their data platforms to ensure automatic report analysis.<br><br>Stakeholders and time commitment: Radiologists and residents: ~1.5 hours/week (validating NLP model outputs and AI sensitivity adjustments), innovation lead: ~2 hours/week (coordinating AI validation, dashboard development, and system adoption), IT specialists: ~3 hours/week (adjusting technical infrastructure for report integration and workflow automation), ScreenAI engineers (dedicated time for NLP model development and dashboard creation).	*Adapting*<br><br>● Adjusted internal technical systems and connections with the AI solution for seamless data communication and report integration back into the workflow.	*Reconfiguring*<br><br>● Introduced the NLP model, creating a major change in validation and establishing ground truth, which established a new paradigm for monitoring and validation.

**Step 5 (early 2020 - early 2021): Advanced PACS integration**<br><br>● Collaboration: ScreenAI, Medica, and the PACS vendor collaborated to integrate AI application results into the PACS worklist and reporting system.<br>● Learning and Integration: With scripts from the PACS vendor, Medica learned to integrate future AI applications, including communicating with the PACS system's API using the required file formats and result structures.<br><br>Stakeholders and time commitment: Radiologists and residents: ~0.5 hour/week (assessing PACS-integrated AI outputs for clinical usability), innovation lead: ~2 hours/week (aligning PACS integration strategy with hospital needs), IT specialists: ~4 hours/week (implementing PACS scripts, testing integration workflows), PACS vendor engineers (dedicated time for developing API scripts and ensuring compatibility).	Reconfiguring<br><br>● Gained an understanding of the PACS system and engineered proper outputs for integration.<br>● Rethought workflows, including filtering worklists and automating reporting processes.	Reconfiguring<br><br>● Redesigned AI application outputs to determine essential results for integration.<br>● Adjusted their holistic view on implementing the AI application in other medical institutions.
**Step 6 (early 2022 - onward): Setting up a quality system and introduction of a new case (incidental pulmonary embolisms on chest CT)**<br><br>● Quality System Development: ScreenAI developed a quality system (using real-time monitoring) to flag mismatches between radiologists and report labels. Notifications of discrepancies between AI and human observations were checked by an independent radiologist. When AI was suspected to be correct, ScreenAI notified the radiologist via email, though there was some delay in notifications, noted as a limitation.<br>● Reference Site: Medica acted as a reference site for investors and other hospitals to obtain information about ScreenAI. Radiologists provided feedback, leading to changes. ScreenAI released a new model, and Medica tested the detection of pulmonary embolisms on chest CT using the same quality system.<br>● Implementation Improvements: Medica improved implementation as CT processing increased system workload. ScreenAI and Medica sought solutions to reduce workload, such as moving parts of the solution to the cloud and prioritizing chest X-rays over CT to avoid delays.<br><br>Stakeholders and time commitment: Radiologists and residents: 0.5 hours/week (reviewing AI-flagged discrepancies, providing feedback),<br>innovation lead: ~2 hours/week (coordinating implementation, acting as a reference site),<br>IT specialists: ~3 hours/week (adapting infrastructure for real-time monitoring and cloud transition),<br>ScreenAI engineers (dedicated time for expanding quality system functionalities and integrating new modalities).	*Adapting*<br><br>● Adapted the data platform to facilitate access to relevant data for the quality system.<br>● Implemented the notification system (using email) for cases where AI and radiologists disagreed.<br>● Worked on a solution to host both chest X-ray and CT solutions (modifying existing systems).	*Adapting*<br><br>● Extended the use of the monitoring system to create email notifications for radiologists.<br>● Expanded the AI solution to include a new modality (CT).<br>● Worked on a solution to host both chest X-ray and CT solutions (modifying existing systems)

**Step 7 (mid 2024 - onward): Moving to real-time modification and notification (discussions)**<br><br>● Future Functionality Ideation: Initiated discussions on future functionalities of the AI solution to provide real-time feedback on the quality of reporting.<br>● Redefining Architecture: Redesigned the AI solution's architecture to work on real-time modification and notification for radiologists, acting as an active engine to check images and provide immediate feedback on any issues in the radiologist's report.<br>● Workflow Rethinking: Medica rethought the logic and workflow for implementing AI, transitioning it from a passive tool to an active, real-time agent.<br>● Legal and Perception Considerations: Addressed new legal considerations and redefined perceptions of AI's usefulness for radiologists, moving from a passive first reader to an active parallel reader.<br><br>Stakeholders and time commitment: Radiologists and residents: ~0.5 hours/week (conceptual discussions on AI's role in real-time feedback),<br>Innovation lead: ~2 hours/week (coordinating discussions with PACS vendors and stakeholders)<br>IT specialists: ~1 hours/week (exploring technical feasibility of real-time AI modification), ScreenAI engineers (dedicated time for investigating integration possibilities).	*Reconfiguring*<br><br>● Rethought the concept of AI from a passive first reader to an active second reader, changing workflow assumptions.<br>● Investigated integration possibilities with the PACS vendor.	*Reconfiguring*<br><br>● Evaluated possibilities regarding the implementation of these functionalities<br>● Performed investigations on the possible use of such a system at multiple institutions.

Table A3 : Co-creation of Chest CT application with ChestAI

Co-creation step (events)	Medica moves	AI startup moves
**Step 1 (September 2018 - early 2019): Initiating the research and exploratory collaboration**<br><br>● September 2018: ChestAI owners met with Medica during the Medtech's health event to support promising AI startups.<br>● The company presented its "search" function for finding relevant radiological cases based on search entries. The function was unique, but its value proposition needed further exploration.<br>● ChestAI collaborated with medical publishers to offer high-quality medical information coupled with imaging data.<br>● An initial meeting with Medica aimed to explore the potential value for radiologists in using the ChestAI solution.<br>● A first agreement was drafted and signed: ChestAI provided the installation, while Medica offered feedback and independent evaluation of the product (with potential publications and other documents, as well as presentations and oral communications).<br><br>Stakeholders and time commitment: Radiologists and residents: ~0.5 hours/week (providing clinical insights and evaluating AI feasibility), innovation lead: ~2 hours/week (coordinating initial collaboration and defining project scope), legal & compliance officers: ~1 hour/week (reviewing legal and ethical considerations for co-creation), AI startup technical team (time allocated for defining required technical infrastructure and installation).	*Resourcing*<br><br>● Utilized the existing Radiology department setup to explore the AI tool's possibilities, leveraging current knowledge and expertise to test and evaluate the AI solution.	*Resourcing*<br><br>● Provided the tool for use and leveraged the existing product to test its practical value.
**Step 2: (almost the entire year of 2019): The first cloud-based implementation of AI**<br><br>● System Redesign: Medica redesigned their systems and redefined their implementation modes for AI solutions, as this was their first cloud-based application.<br>● Initial Skepticism: Medica's IT department was initially skeptical and had to navigate a new paradigm of data protection assessment (DPA) and security documentation before signing the contract.<br>● Organizational Challenges: It was unclear whom to contact at Medica since this was not a standard software implementation.<br>● Security and Certification: Meetings with Medica's IT security teams revealed that ChestAI needed to obtain specific ISO certification for security and make system changes to ensure secure data handling.<br><br>Stakeholders and time commitment: IT security and compliance teams: ~1 hours/week (assessing security risks, reviewing data protection measures, and ensuring	*Reconfiguring*<br><br>● Shifted to a cloud-based implementation paradigm.<br>● Rethought security and AI implementation using an on-premise gateway with cloud connection.	*Adaptation*<br><br>● Obtained necessary certifications and improved their security framework, expanding technical security measures.

compliance with regulatory requirements). Innovation lead: ~1 hours/week (coordinating discussions between technical teams and hospital leadership to align AI implementation with institutional goals). Legal & compliance officers: ~1 hours/week (reviewing contracts, ensuring adherence to data protection laws, and facilitating risk assessments). ChestAI technical team: Dedicated time for implementing system changes, obtaining specific ISO certification, and addressing security concerns raised by Medica's IT department.		
**Step 3: (late 2019- early 2020): Basic Integration into PACS**<br><br>● Web-based Application Accessibility: The web-based application had to be accessible from within PACS. Medica avoided direct use of the web-based application due to workflow distractions, opting for a URL-based callback mechanism within PACS.<br>● System Modification: Medica modified their system to automatically forward images to the AI solution using scripts and view the results.<br>● Token-based Authentication: ChestAI adjusted their web-based system to automatically recognize and accept PACS users using token-based authentication, eliminating repetitive log-ins.<br><br>Stakeholders and time commitment: PACS administrators: ~1 hour/week (configuring PACS to enable image forwarding and integrating URL-based callbacks). IT security and compliance teams: ~0.5 hours/week (ensuring token-based authentication met security standards). Innovation lead: ~1 hour/week (facilitating coordination between Medica, ChestAI, and PACS administrators). ChestAI technical team: Dedicated time for implementing token-based authentication and adapting the web application for seamless PACS integration.	*Adaptation*<br><br>● Modified the PACS interface to forward data to the ChestAI solution.<br>● Configured token-based authentication in PACS to automatically log PACS users into the ChestAI solution.	*Adaptation*<br><br>● Modified the ChestAI solution to accept token-based authentication.
**Step 4 (late 2019- late 2020): Quantification of lung pathologies**<br><br>● Pilot Implementation Feedback: After the pilot implementation at Medica and similar experiences in other institutions, it became clear that the image-based search functionality offered limited value for specialized radiologists. They found pattern recognition easy but needed quantification of percentage lung coverage for clinical relevance.<br>● Redefinition of Value Proposition: This led to redefining the AI solution's value proposition, focusing on quantification and detection of anatomical and pathological issues.<br>● COVID-19 Needs: The need for quantifying lung coverage of different pathologies accelerated implementation due to the COVID-19 pandemic, making quantitative findings integral.	*Resourcing*<br><br>● Offered feedback based on medical expertise.	*Reconfiguring*<br><br>● Redesigned the AI solution to include image quantification functionality, rethinking the user interface, and implementing different logics in the software.

● Ongoing Feedback: Medica provided feedback and actively participated in the AI solution's redesign.<br><br>Stakeholders and time commitment: Radiologists and residents: ~1 hour/week (providing clinical feedback on quantification relevance and usability). Innovation lead: ~1 hour/week (aligning AI development with clinical needs and pandemic-related priorities). ChestAI technical team: Dedicated time for modifying the AI model to focus on lung pathology quantification.		
**Step 5 (mid 2020- early 2021): Deep PACS integration**<br><br>● Limitations and Rethinking Integration: Noticing the limitations of having AI results in the web application, Medica rethought the integration strategy into an advanced integration, redesigning the AI solution, interfaces, and part of PACS for seamless integration.<br>● Technical Collaboration: Medica worked with the PACS provider to implement the required technical changes, integrating AI results within the worklists and reporting system, including an automated accept/reject module for reporting pulmonary nodules.<br>● Data and Communication Changes: ChestAI fundamentally changed the data and communication of the AI solution to directly interact with PACS.<br><br>Stakeholders and time commitment: PACS administrators and provider: ~1.5 hours/week (implementing deep integration, modifying PACS interfaces, and enabling AI result embedding).<br>Radiologists: ~0.5 hours/week (validating AI output within PACS workflow and assessing reporting efficiency).<br>Innovation lead: ~1 hour/week (coordinating between Medica, PACS provider, and ChestAI to ensure seamless integration). ChestAI technical team: Dedicated time for redesigning data flow and communication protocols to integrate directly with PACS.	*Reconfiguring*<br><br>● Reconfigured PACS for ChestAI and future AI integrations.<br>● Created report templates that could be prefilled with AI results.<br>● Configured the accept/reject module in the PACS system.	*Reconfiguring*<br><br>● Redesigned the ChestAI solution to provide the correct output.<br>● Rethought the value proposition of the ChestAI solution, focusing more on nodule detection and quantification and considering other customers.
**Step 6 (late 2021 -early 2022): Adjusting and fine-tuning in practice**<br><br>● Clinical Practice Feedback: Medica worked with the new solution in clinical practice, solicited feedback from radiologists, and discovered the application was too sensitive to medically irrelevant findings (e.g., reporting cases with <1% pathological lung tissue). After discussions with radiologists, a tentative agreement was reached that such findings should not be reported.<br>● Requested Adjustments: Other adjustments and adaptations were requested through implementation and testing by radiologists. The aim was to find consensus while keeping functionalities generalizable for implementation at different institutes with varying reporting requirements.<br>● Adaptations by ChestAI: ChestAI adapted their solutions to provide different functionalities and made	*Adapting*<br><br>● Learned what works in practice and what to ask/expect from the AI solution in terms of presenting results.<br>● Found group consensus and modified existing reporting templates.	*Adapting*<br><br>● Introduced new functionalities to configure the reporting of AI results.<br>● Adjusted the reporting and interface of the solution to better align with the needs of radiologists.

them configurable to satisfy requirements at different sites.<br><br>Stakeholders and time commitment: Radiologists: ~1 hour/week (providing clinical feedback on AI sensitivity and reporting thresholds). Innovation lead: ~0.5 hours/week (coordinating discussions to balance generalizability and site-specific needs).<br>ChestAI technical team: Dedicated time for refining AI sensitivity, implementing configurability, and adapting functionalities to different institutional requirements.		
**Step 7 (late 2022- late 2023): Timeline and risk-scoring solution**<br><br>● Feedback on Temporal Analysis: After implementing the quantification and detection solution, Medica and other clients provided feedback on the need for performing temporal analysis of multiple cases, particularly for tracking changes in pulmonary nodules over time.<br>● Proposition Requirements: This required analyzing and comparing multiple image sets, including prior radiological exams. A radical redesign of the AI application was necessary to build in new logic for connecting multiple imaging exams taken at different time points.<br>● Data Organization Challenges: Medica faced challenges in organizing and storing data, as automated prefetching and retrieval of prior cases became essential.<br>● Strategies to Overcome Difficulties: Various strategies were proposed to overcome difficulties with automated forwarding. One solution involved manually forwarding cases the day before to ensure all results were available when the patient underwent imaging the next day.<br>● Incorporation of Risk Scoring: In addition to quantification, the new ChestAI solution incorporated technology from another vendor to perform risk scoring of pulmonary nodules. Medica validated this risk scoring on independent data sets to add value in clinical practice.<br><br>Stakeholders and time commitment:<br>Radiologists: ~0.5 hour/week (validating temporal analysis and risk-scoring utility in clinical practice).<br>PACS administrators: ~1 hour/week (configuring automated prefetching and retrieval of prior cases).<br>Innovation lead: ~0.5 hours/week (coordinating workflow integration and aligning AI logic with clinical needs).<br>ChestAI technical team: Dedicated time for redesigning AI to support multi-timepoint analysis and integrating risk-scoring technology. External AI vendor: Collaborative effort (providing risk-scoring technology and supporting validation on independent datasets).	*Adapting (actively)*<br><br>● Implemented manual forwarding of prior exams the day before patient arrival.<br>● Adapted clinical workflows where radiographers prepare AI results for radiologists.<br>● Validated and incorporated pulmonary nodule cases into Medica's workflow.<br>● Investigated possibilities to automate pre-processing of CT images.	*Reconfiguring*<br><br>● Redesigned the AI solution to incorporate pulmonary risk scoring.<br>● Extended functionality to include temporal analysis and timeline views for visualizing changes over time.<br>● Created a new value proposition for ChestAI to diagnose and characterize pulmonary nodules in a population screening setting.

**Step 8 Automating forwarding of priors (early 2024 - onward)**<br><br>• Manual Forwarding to Automation: With manual forwarding plans in place, Medica collaborated with ChestAI and their PACS vendor to explore possibilities for automatically forwarding prior cases for pre-processing the day before the patient arrives. Strategy development and implementation planning are still ongoing.<br><br>Stakeholders and time commitment: PACS administrators and providerr: ~1 hour/week (developing and testing automated forwarding solutions).<br>Innovation lead: ~0.5 hours/week (coordinating discussions between Medica, ChestAI, and the PACS vendor). ChestAI technical team: Dedicated time for implementing automated pre-processing and ensuring compatibility with PACS workflows.	*Reconfiguring*<br><br>• Conducted a technical deep dive into possibilities for automating priors to the ChestAI solution.<br>• Proposed several technical solutions and investigated their feasibility.	*Reconfiguring*<br><br>• Leveraged experience from other medical institutions to consider alternative methods for automating the forwarding of priors using different technical capabilities that are not yet fully developed.
